# Characteristics of the *Vasa* Gene in *Silurus asotus* and Its Expression Response to Letrozole Treatment

**DOI:** 10.3390/genes15060756

**Published:** 2024-06-08

**Authors:** Miao Yu, Fangyuan Wang, Muzi Li, Yuan Wang, Xiangzhe Gao, Hanhan Zhang, Zhenzhu Liu, Zhicheng Zhou, Daoquan Zhao, Meng Zhang, Lei Wang, Hongxia Jiang, Zhigang Qiao

**Affiliations:** 1 Engineering Technology Research Center of Henan Province for Aquatic Animal Cultivation, Observation and Research Station on Water Ecosystem in Danjiangkou Reservoir of Henan Province, College of Fisheries, Henan Normal University, Xinxiang 453007, China; wypajk@163.com (F.W.); 17639405479@163.com (M.L.); m19712555615@163.com (Y.W.); gaoxiangzhe37@163.com (X.G.); 15225938165@163.com (H.Z.); zhenzhuliu1205@163.com (Z.L.); zhouzc2025@163.com (Z.Z.); mzhangshou@163.com (M.Z.); wanglei201912@htu.edu.cn (L.W.); jianghongxia@htu.cn (H.J.); 13503800008@126.com (Z.Q.); 2 Yiluo River Aquatic Biology Field Scientific Observation and Research Station in the Yellow River Basin of Henan Province, Lushi, Sanmenxia City 472200, China; zhaodaoquan@163.com

**Keywords:** *Silurus asotus*, *vasa*, letrozole, gonadal development, gene expression

## Abstract

The identification and expression of germ cells are important for studying sex-related mechanisms in fish. The *vasa* gene, encoding an ATP-dependent RNA helicase, is recognized as a molecular marker of germ cells and plays a crucial role in germ cell development. *Silurus asotus*, an important freshwater economic fish species in China, shows significant sex dimorphism with the female growing faster than the male. However, the molecular mechanisms underlying these sex differences especially involving in the *vasa* gene in this fish remain poorly understood. In this work, the *vasa* gene sequence of *S. asotus* (named as *Savasa*) was obtained through RT-PCR and rapid amplification of cDNA end (RACE), and its expression in embryos and tissues was analyzed using qRT-PCR and an in situ hybridization method. Letrozole (LT) treatment on the larvae fish was also conducted to investigate its influence on the gene. The results revealed that the open reading frame (ORF) of *Savasa* was 1989 bp, encoding 662 amino acids. The SaVasa protein contains 10 conserved domains unique to the DEAD-box protein family, showing the highest sequence identity of 95.92% with that of *Silurus meridionalis*. In embryos, *Savasa* is highly expressed from the two-cell stage to the blastula stage in early embryos, with a gradually decreasing trend from the gastrula stage to the heart-beating stage. Furthermore, *Savasa* was initially detected at the end of the cleavage furrow during the two-cell stage, later condensing into four symmetrical cell clusters with embryonic development. At the gastrula stage, *Savasa*-positive cells increased and began to migrate towards the dorsal side of the embryo. In tissues, *Savasa* is predominantly expressed in the ovaries, with almost no or lower expression in other detected tissues. Moreover, *Savasa* was expressed in phase I–V oocytes in the ovaries, as well as in spermatogonia and spermatocytes in the testis, implying a specific expression pattern of germ cells. In addition, LT significantly upregulated the expression of *Savasa* in a concentration-dependent manner during the key gonadal differentiation period of the fish. Notably, at 120 dph after LT treatment, *Savasa* expression was the lowest in the testis and ovary of the high concentration group. Collectively, findings from gene structure, protein sequence, phylogenetic analysis, RNA expression patterns, and response to LT suggest that *Savasa* is maternally inherited with conserved features, serving as a potential marker gene for germ cells in *S.asotus*, and might participate in LT-induced early embryonic development and gonadal development processes of the fish. This would provide a basis for further research on the application of germ cell markers and the molecular mechanisms of sex differences in *S. asotus*.

## 1. Introduction

The gene *vasa* encodes an ATP-dependent RNA helicase of the DEAD (Asp-Glu-Ala-Asp)-box family protein, initially discovered in *Drosophila* as a maternal-effect gene [1]. Subsequently, *vasa* homolog genes have been identified in various species including mice (*Mus spretus*) [2], *Xenopus* [3], silkworms (*Bombyx mori*) [4], humans (*Homo sapiens*) [5], and the sea urchin (*Anthocidaris crassispina*) [6]. In fish, the *vasa* gene was first characterized in zebrafish (*Danio rerio*) [7,8], followed by medaka (*Oryzias latipes*) [9,10], tilapia (*Oreochromis niloticus*) [11], gibel carp (*Carassius auratus gibelio*) [12], southern catfish (*S. meridionalis*) [13], discus fish (*Symphysodon haraldi*) [14], black carp (*Mylopharyngodon Piceus*) [15], and so on.

The RNA helicase Vasa has a significant impact on various mRNA metabolic processes [16]. Additionally, the *vasa* gene is known to be crucial in gametogenesis [8,17,18] and is widely recognized as a germ cell biomarker across different species [5,8,12,19,20,21]. In *Drosophila*, *vasa* knockout results in abnormal primordial germ cell (PGC) differentiation and oocyte maturation failure, as well as the failure of localized RNAs to accumulate in oocytes [22,23]. In zebrafish, loss of the germ-line stem cells causes all *vasa*-mutant fish to form an immature testis [18,24], and *vasa* knockdown inhibits RNA translation without affecting germline establishment [25]. In medaka, the *vasa* gene is necessary for PGCs migration [26]. These findings highlight the crucial role of *vasa* in RNA metabolic and germ cell development across species.

*S. asotus*, known as the Yellow River catfish, is a freshwater benthic carnivorous fish belonging to the Siluriformes order, Siluridae family, *Silurus* genus. It is widely distributed in various water systems in China except for Xinjiang and the Qinghai–Tibet Plateau and is an important indigenous economic fish species. To date, the research on *S. asotus* has covered various aspects such as its basic biology, breeding techniques, reproductive physiology, nutrition and digestive physiology, transcriptomics, and disease prevention and control [27,28,29,30]. However, reports on its germ cells and sex-related mechanism including the role of *vasa* are lacking. Given the significant allometric growth phenomena in this species with females growing faster than males, investigating the *vasa* gene might provide essential data for future research on *S. asotus.*

There have been numerous studies on fish sex reversal, indicating that the sex of fish is highly flexible and not solely determined by genetic factors, but it is also influenced by external environmental conditions. Manipulating these environmental factors before or during early sex differentiation stages would attribute to artificial control of fish sex [31,32,33]. Exogenous induction, for instance, the aromatase inhibitor LT treatment, is a common method in artificial sex control of fish. LT, a benzotriazole derivative, is known for its high selectivity in inhibiting aromatase and increasing endogenous gonadotropin secretion. Successful experiments using LT-induced fish sex reversal have been conducted in some fish species, such as *Epinephelus akaara*, *Nibea albiflora,* and *S. asotus* [34,35,36]. And in *S. asotus*, our team has achieved 100% male fish by inducing normal larvae with LT [36]. Nevertheless, the molecular mechanisms underlying LT-induced sex reversal in this fish species, as well as changes in the expression of the germ cell marker *vasa* remain unclear. More studies should be carried out to understand it.

Taken together, the present study aimed to clone and identify the *vasa* ortholog in *S. asotus*, named as *Savasa*. This study further examined the expression pattern of *Savasa* during embryogenesis and gametogenesis using qRT-PCR, WISH, and FISH techniques. Additionally, *S. asotus* larvae were subjected to LT treatment to observe the response of the *vasa* gene to the treatment. The results would provide a foundation for future research and application on labeling, isolation, and transplantation of embryonic and gonadal germ cells, potentially contributing to advancements in single-sex breeding strategies for the aquaculture species of *S. asotus*.

## 2. Materials and Methods

### 2.1. Animals Treatment and Samples Collection

Healthy adult *S. asotus* were obtained from the recirculation aquaculture system at the College of Fisheries, Henan Normal University, and acclimated for one week before the experiment. The fish were fed with commercial feed twice daily. Tissues including the brain, heart, liver, kidney, spleen, intestines, testis, and ovary from six of the fish were collected for gene clone and expression analysis. Fertilized eggs were obtained via artificial fertilization from two parental fish. Embryos were cultured in the recirculation aquaculture system at 24 ± 1 °C with a 14 h light to 10 h darkness photoperiod. Embryos at various stages were collected, including the 2-cell, 4-cell, 8-cell, 16-cell, 32-cell, multi-cell, blastula, gastrula, neurula, eye vesicle, tail-bud, muscle effect, and heart-beating and hatching stages. Samples for qRT-PCR analysis were supplemented with Sample Protector for RNA/DNA (TaKaRa, Kusatsu, Japan) and stored at −80 °C until RNA extraction. Samples for in situ hybridization were fixed with 4% paraformaldehyde (PFA). All procedures adhered to the guidelines approved by the Academic Committee of Henan Normal University (HNSD-2024-08BS-25).

### 2.2. RNA Extraction and cDNA Synthesis

Total RNA was extracted from either pooled defined tissues or 30–40 embryos at specific stages of *S.asotus* using the TRIzol reagent (Invitrogen, Waltham, MA, USA). Subsequently, the quality of the total RNA was assessed through 1% agarose gel electrophoresis, while the concentration and purity were determined through a *N_ANO_D_ROP_ 2000* spectrometer (Thermo Scientific, Waltham, MA, USA). The first-strand cDNA synthesis was carried out using 1 μg total RNA by the PrimeScript^TM^ RT reagent kit with gDNA Eraser (TaKaRa, Kusatsu, Japan).

### 2.3. Isolation of Savasa cDNA Sequence

Primers were designed based on conserved sequences of striped catfish (XM_026941979), channel catfish (NM_001329311), and yellow catfish (XM_027141183) in the NCBI database (Table 1) using Primer 5.0 software. The open reading frame (ORF) was confirmed through PCR amplification using ovary cDNA with the following conditions: pre-denaturation of 3 min at 94 °C, 32 cycles of 20 s at 94 °C, 30 s at 57 °C, and 2 min at 72 °C. The resulting PCR product was then cloned into a pMD 19-T vector (TaKaRa, Kusatsu, Japan) and validated through sequencing. Subsequently, two specific primers of Sa-v-GSP and Sa-v-NGSP were designed for amplification of the 3ʹ untranslated region (UTR) in conjunction with UPM and NUP primers. The procedure was performed for 3 min at 95 °C, followed by 5 cycles of 95 °C for 20 s, 64 °C for 30 s, and 72 °C for 2 min; 10 cycles of annealing at 62 °C; and 20 cycles of annealing at 60 °C and then 72 °C for 10 min. PCRs were performed on a ProFlex PCR system (Life Technologies, Carlsbad, CA, USA).

### 2.4. Sequence Analysis

The cDNA sequence assembly and protein sequence alignment was performed using DNAMAN 6.0 software (Lynnon Corporation, San Ramon, CA, USA). BLAST analysis was conducted on the NCBI database (http://blast.ncbi.nlm.nih.gov/Blast.cgi, accessed on 1 June 2022). A phylogenetic tree was constructed through the neighbor-joining algorithm on the MEGA 6.0 program.

### 2.5. qRT-PCR Analyses

Expression patterns of *Savasa* in different developmental embryos and adult tissues were performed by using quantitative real-time PCR (qRT-PCR) with a SYBR^®^ Premix DimerEraser^TM^ kit (TaKaRa, Kusatsu, Japan) on a ViiA^TM^ 7 DxReal-Time PCR System (ABI, Foster City, CA, USA). The thermal cycling conditions included 95 °C for 10 s, followed by 40 cycles at 59 °C for 10 s, and 72 °C for 10 s, then 95 °C for 1 s and 65 °C for 15 s to 95 °C (0.11 °C/s), with a finalization step at 40 °C. The primers used are listed in Table 1. Each sample was run in triplicate with an internal control in a 96-well plate. A melting curve analysis and direct sequencing of qRT-PCR products were conducted to confirm primer specificity. *β*-*actin* was used as the reference gene. The relative expression level of *Savasa* was normalized against the expression level in the embryos of the heart-beating stage or in the tissues of brain using the 2^−ΔΔCt^ method [37].

### 2.6. In Situ Hybridization

The whole-mount in situ hybridization (WISH) procedure was performed following previously published methods with slight adjustments [38]. In summary, embryos at different developmental stages and specific tissues were fixed in 4% PFA in 0.1 M phosphate-buffered saline (PBS, pH 7.4) at 4 °C for 24 h. A 981 bp fragment of *Savasa*, which includes the *vasa* domain, was sub-cloned into the pCS2+ vector. The vector was linearized using *Eco*RIor *Xba*I, and sense and anti-sense RNA probes were synthesized from the SP6 or T7 promoter using the digoxigenin (DIG) RNA labeling kit (Roche, Basel, Switzerland). The probes underwent treatment with RNase-free TURBO DNase (Ambion, Austin, TX, USA) and were purified accordingly. The embryos were then subjected to hybridization with the sense or anti-sense probes at 65 °C for 12 h. The hybridization signal was visualized by staining with the anti-DIG antibody-conjugated alkaline phosphatase and nitroblue tetrazolium/5-bromo-4-chloro-3-indolyl phosphate (NBT/BCIP, Sigma, St. Louis, MO, USA) as the chromogenic substrate. The hybridizations were observed, and images were captured using a stereomicroscope (LEICA, DFC550, Wetzlar, Germany). Additionally, fluorescence in situ hybridization (FISH) was performed as described by Xu et al. [12] and Yu et al. [38]. Paraffin-embedded sections of the testis and ovary (5 μm thickness) were prepared, and the sense and anti-sense RNA probes were utilized as previously mentioned. The signals were detected by staining with Cy3 and 4′,6-diamidino-2-phenylindole (DAPI) as the substrate, and the FISH experiment was carried out by Wuhan Servicebio Technology Co., Ltd. (Wuhan, China).

### 2.7. LT Treatment

Larvae were treated with LT starting from 8 days post hatching (dph). Three treated groups (20 mg/kg, 35 mg/kg, and 50 mg/kg) and one control group were established, each with 3 replicates and 70 larvae per replicate. The control group was raised under normal conditions without LT treatment. At 40 dph, all groups were switched to normal feed (same as control) for continued feeding. LT was first dissolved in 95% ethanol to create a mother liquor with a concentration of 1 mg/L, which was then mixed with feed at the specified concentrations. Gonad samples were collected from the four groups on the 8, 16, 22, 24, 40, 60, 90, and 120 dph, with 6 fish randomly selected from each group. Fish with a total length of less than 2 cm were entirely preserved in RNA/DNA preservation solution, while those measuring 2–8 cm had the trunk part containing the gonads stored in the solution. Fish longer than 8 cm, where the gonads could be identified and separated, had only the gonadal tissues preserved in the RNA/DNA preservation solution.

### 2.8. Statistical Analysis

All the qRT-PCR data were presented as means ± S.D. (*n* = 3). A one-way analysis of variance (one-way ANOVA) was performed to assess the differential expression levels of the *Savasa* gene using SPSS 23.0 (SPSS, Chicago, IL, USA). Statistical significance was determined at *p* < 0.05.

## 3. Results

### 3.1. Identification and Molecular Characterization of Savasa

**Figure 1:**
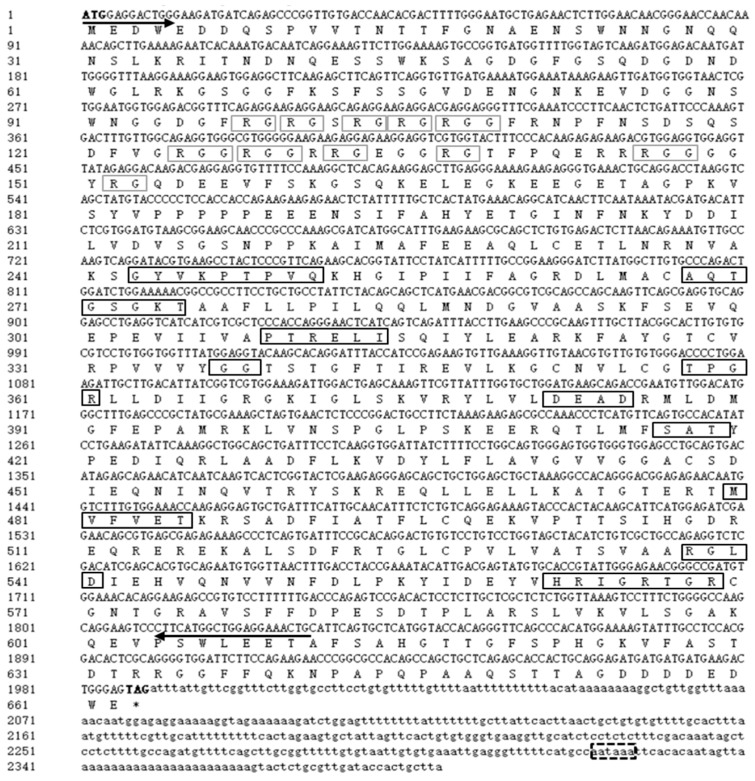
Nucleotide and deduced amino acid sequence of *Savasa.* The start codon (ATG) and stop codon (TAG) are highlighted in bold. The ORF is shown in uppercase letters, whereas the 3′ untranslated regions are indicated in lower case. The amino acid sequences are displayed underneath the ORF using single capital letter codes. The putative polyadenylation signal aataaa is in a black dashed box. The primer sequences for *Savasa* ORF synthesis are indicated by horizontal arrows. The ten conserved motifs for the DEAD protein family are highlighted in black boxes. The RG and RGG repeats in the N-terminal region are noted in gray boxes.

**Figure 2:**
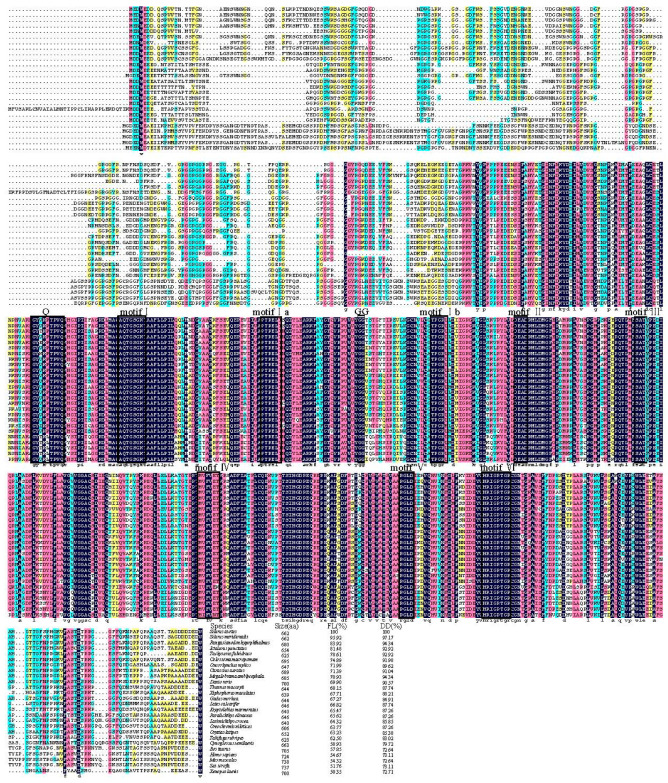
Multiple sequence alignment of Vasa proteins. Different colored letters indicate their degree of conservatism in different sequences. Positions of identical residues are indicated in black. Ten conserved motifs of the DEAD protein family are highlighted in black frame. The species’ names, amino acid length, and the percentage of identities for full length (FL) and DEXDc domain (DD) in other species homologues to that of SaVasa are represented at the end of alignment.

The cDNA sequence of *Savasa* was identified and characterized from the ovarian cDNA library. *Savasa* consists of a 1989 bp ORF that is predicted to code a protein of 662 amino acid (aa) residues with an estimated molecular mass of 72.28 kDa and a 460 bp 3′ untranslated region (UTR) (Figure 1). The predicted protein contains a representative DEXDc domain at aa positions 243–454 and HELICc domain at aa positions 490–571. Within these domains, there are ten conserved motifs of the DEAD-box protein family, including xYxxPTPVQ(Q), AQTGSGKT(I), PTRELI(Ia), TPGR(Ib), DEAD(II), SAT(III), MVFVET(IV), RGLD(V), HRIGRTGR(VI), and GG-enriched sequences. Additionally, there are seven arginine-glycine (RG) and four arginine-glycine-glycine (RGG) repeat sequences at the N-terminus (Figure 1). These structures exhibit similarity to other Vasa proteins based on sequence alignment. Furthermore, sequence alignment of the Vasa protein showed high identity between the full-length and DEXDc domain length of the SaVasa and Vasa proteins from *Silurus meridionalis* (95.92%, 97.17%), *Pangasianodon hypophthalmus* (83.92%, 94.34%), *D. rerio* (69.90%, 90.57%), *Xenopus laevis* (50.35%, 72.71%), and *H. sapiens* (54.67%, 73.11%), respectively (Figure 2).

**Figure 3:**
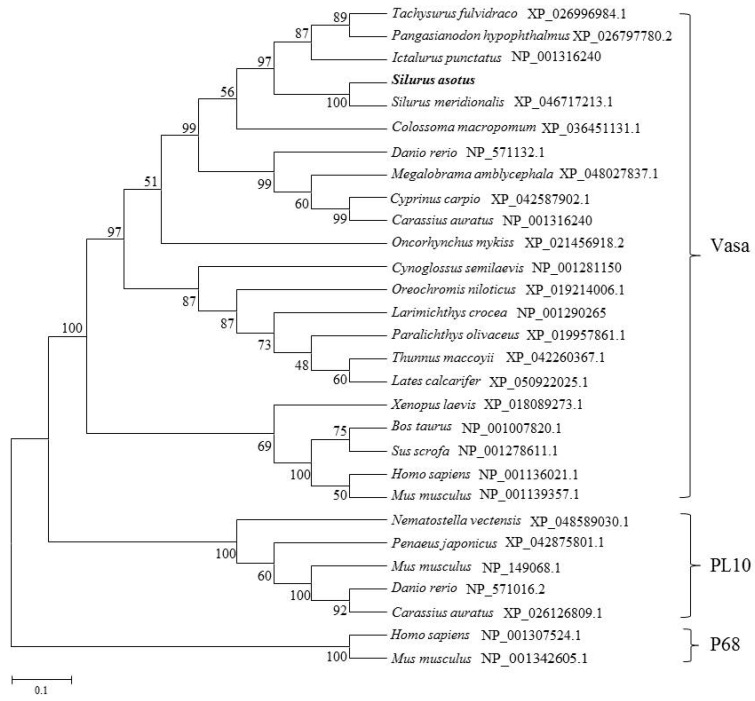
Phylogenetic tree analysis of Vasa proteins. The branching between *S. asotus* and others was deduced by MEGA 6.0 software using Poisson Correction distance based on the neighbor-joining method with 1000 bootstrap replicates. Numbers next to the branches suggest bootstrap values.

The phylogenetic tree was constructed using the neighbor-joining algorithm to illustrate the phylogenetic relationship between SaVasa and its counterparts in other species (Figure 3). Within the DEAD-box protein families, there were three distinct groups: the Vasa subfamily, the PL10 subfamily, and the P68 subfamily. SaVasa was clustered with the Vasa subfamily members found in fish and land vertebrates within the DEAD-box family, while being separate from the PL10 and P68 subfamilies (Figure 3). These results indicated that the *vasa* sequence we cloned here should be the homolog of the *Vasa* gene, and as such, we have designated it as *Savasa*.

### 3.2. Maternal and Gonad-Predominant Expression of Savasa

**Figure 4:**
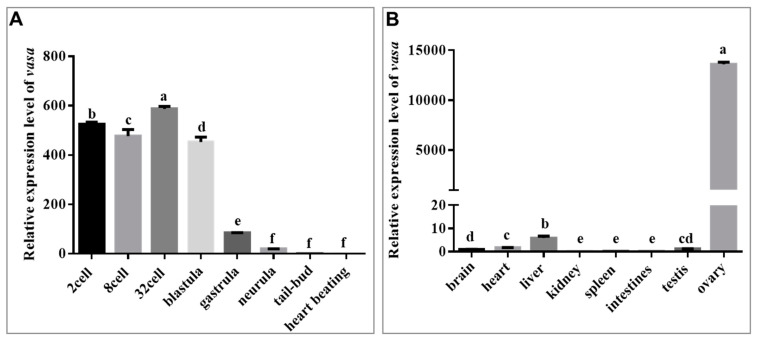
The spatio-temporal expression levels of *Savasa* using qRT-PCR analysis. (**A**) The relative expression of *Savasa* at different developmental stages. All samples were normalized against the heart beating stage. (**B**) The relative expression of *Savasa* in different adult tissues. The transcripts are predominant in the ovary. All samples were normalized against the brain. The *β-actin* gene was used as the reference gene. Data are shown as mean ± SD (*n* = 3). Different lowercase letters indicate significant differences of *Savasa* expression levels during different development stages or tissues, and statistical difference was set at *p* < 0.05.

The mRNA expression pattern of *Savasa* was initially assessed in early embryos using qRT-PCR. Results indicated that *Savasa* mRNA was highly expressed from the two-cell stage to the blastula stage, suggesting its maternal inheritance. Subsequently, there was a gradual decrease on its expression from gastrula to the heart-beating stage (Figure 4A). Furthermore, qRT-PCR analyses revealed that there was almost no expression of *Savasa* transcripts in adult tissues like those of the kidney, spleen, and intestines, with lower expression levels in the liver, heart, brain, and testis, while showing predominant expression in the ovary, suggesting main localization of *Savasa* mRNA in the gonads (Figure 4B).

### 3.3. Distribution of Savasa mRNA in the Developmental Embryos

**Figure 5:**
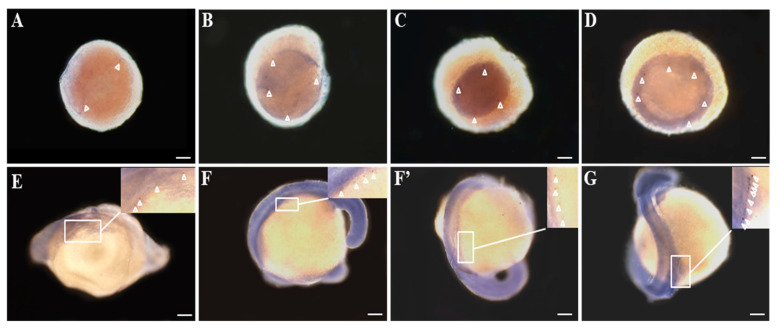
Distribution of *Savasa* mRNA in the developmental embryos by WISH analysis. The positive cells with anti-sense *vasa* probe hybridization were stained purple (**A**–**G**) at different stages: (**A**) 2-cell; (**B**) 4-cell; (**C**) blastula; (**D**) gastrula; (**E**) eye vesicle stage; (**F**,**F**′) muscle effect stage; (**G**) hatching stage. Scale bars = 200 μm. WISH results revealed that the *Savasa* mRNA-positive signals were distinct from the 2-cell stage to hatching stage, presented by asterisk arrowheads.

The spatial-temporal expression of *Savasa* was additionally analyzed by WISH during embryogenesis. *Savasa* mRNA-positive signals consistently present at all the examined embryonic stages, showing variations in numbers and locations (Figure 5A–G). The positive signals also indicate the migration route of PGCs in *S. asotus*. Briefly, at the two-cell stage, *Savasa* signals clustered at both ends of the first cleavage furrow (Figure 5A). During the four-cell and blastula stage, *vasa* mRNA was expressed in four clusters along the cleavage furrow (Figure 5B) or in the marginal region of the blastomere (Figure 5C). During the gastrula stage, the number of *Savasa*-positive cells increased, with signals migrating from the marginal to the internal region of the embryo (Figure 5D). Subsequently, at the eye vesicle stage, *Savasa*-positive cells became linear and migrated towards the dorsal side (Figure 5E). Then these cells continuously migrated along the dorsal side, displaying two rows on either side of the embryo axis from the muscle effect stage (Figure 5F,F′). By the hatching stage, *Savasa*-positive cells further increased in number, forming bilateral rows on the dorsolateral region near the caudal segments (Figure 5G). This PGCs migration route was similar to that in zebrafish [39]. In contrast, no visible hybridization signals were detected using the sense probe throughout embryogenesis (Supplementary Data, Figure S1).

### 3.4. Germ Cells-Specific Expression of Savasa in Adult Gonads

**Figure 6:**
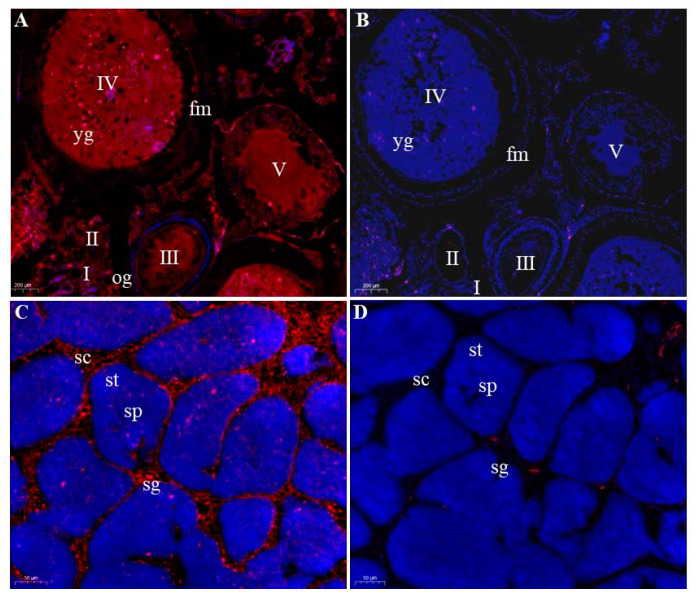
Distribution of *Savasa* mRNA in the gonads by FISH. Ovarian and testicular cross sections stained for *Savasa* mRNA (red) and nucleus with dye DAPI (blue) were analyzed by microscopy. (**A**) the anti-sense probe of *Savasa* mRNA in the ovary; (**B**) the sense probe of *Savasa* mRNA in the ovary; (**C**) the anti-sense probe of *Savasa* mRNA in the testis; (**D**) the sense probe of *Savasa* mRNA in the testis. The sense probe did not show any detectable signals. Og, oogonia; fm, follicular membrane; yg, yolk granules; I–V, stages of oocytes; sg, spermatogonia; sc, spermatocytes; st, spermatids; sp, sperm. The scale bars are 200 μm in the ovary, and 50 μm in the testis.

Further examination in the spatial expression of *Savasa* mRNA in the gonad was detected by FISH with the anti-sense *vasa* DIG probe. The study discovered that high expression of *Savasa* fluorescence signals was in oogonia and primary oocytes from stages I to V (Figure 6A), with no fluorescent signals from the sense probe detected in the ovary (Figure 6B). These results align with qRT-PCR data, indicating a specific expression of *Savasa* in the ovarian germ cells and its role in oogenesis.

We also examined the expression of *Savasa* mRNA in the adult testis. The result noted that *Savasa* mRNA was only detected in germ cells of spermatogonia and spermatocytes but not in spermatids and sperm (Figure 6C), suggesting its potential rule in early spermatogenesis. No signals from the sense probe were detected in the testis (Figure 6D). The FISH results confirmed the specific expression of *Savasa* mRNA in germ cells during gametogenesis.

### 3.5. Response of Savasa Expression to Letrozole Treatment

**Figure 7:**
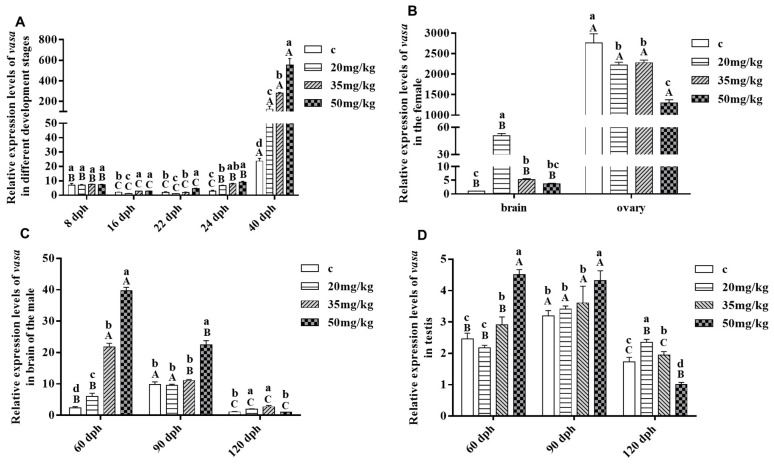
Response of *Savasa* expression levels to different concentrations of Letrozole. (**A**) Different developmental stages, (**B**) brain and ovary of the female at 120 dph, (**C**) brain of the male, (**D**) testis. Bars represent the mean ± S.D. (*n* = 3). Different lowercase letters indicate significant differences of *Savasa* expression levels in different LT concentrations at the same developmental stage or tissue. Different capital letters indicate significant differences in the same LT concentration at different developmental stages or tissues (*p* < 0.05).

Following treatment of 8 dph larvae with different concentrations of LT, the expression levels of *Savasa* were found to vary across different developmental stages and tissues, as shown in Figure 7. Specifically, there was a notable elevation in *Savasa* expression levels along with the increasing concentrations of LT from 8 to 40 dph of the larvae, peaking at the concentration of 50 mg/kg at 40 dph (Figure 7A). Notably, in 120 dph of females, the overall expression level of *Savasa* was significantly higher in the ovary compared to the brain, and its expressions at 20 mg/kg and 35 mg/kg exceeded the control in the brain, while its levels at all LT treated groups were lower than the control in the ovary, particularly in the 50 mg/kg group (Figure 7B). In the brain of the male fish, *Savasa* expression levels raised significantly with the rising LT concentrations at 60 dph, its expression only in the 50 mg/kg group increased significantly compared to the control at 90 dph, and its levels in both of the 20 mg/kg and 35 mg/kg groups were higher than the control at 120 dph (Figure 7C). Just as the figure shows, the overall *Savasa* expression level at 120 dph was lower than those at 60 and 90 dph. In the testis of the male fish, *Savasa* expression significantly increased in the 35 mg/kg and 50 mg/kg groups at 60 dph, only elevated significantly in the 50 mg/kg group at 90 dph, and increased significantly in the 20 mg/kg and 35 mg/kg groups while decreasing significantly in the 50 mg/kg group at 120 dph (Figure 7D). Furthermore, comparing with the control, the expression trends of *Savasa* in the ovary were different from those in the testis at 120 dph, showing different responses of *vasa* to LT and indicating diverse functions in different sex of fish in *S.asotus*.

## 4. Discussion

The study detailed the cDNA sequence, expression patterns including the PGC migration route, and subcellular localization of *Savasa* in multiple embryonic developmental stages and tissues especially in the ovary and testis, thus identifying *vasa* as a germ cell marker in *S. asotus* for the first time. Additionally, the effects of LT on Savasa expression in different developmental stages of the juvenile fish and tissues in female and male fish were detected. These investigations provided basic information on *Savasa* gene.

### 4.1. Identification of Savasa

Savasa was designated as the Vase ortholog due to its conserved features. The gene structure of Savasa exhibited a high sequence identity of 95.92% at full-length and 97.17% at the DEXDc domain compared to the *vasa* gene in *Silurus meridionalis*. It shared similarities with other Vasa homologs within the DEAD-box protein family, exhibiting at least eight characteristic motifs of Vasa proteins [40,41]. Notably, the N-terminal region displayed rich RG and RGG repeats, similar to Vasa homologs in other teleosts such as the grass carp, *Drosophila*, and the rainbow trout (*Oncorhynchus mykiss*) [42,43,44]. As known, there are three subfamilies of Vasa, PL10, and P68 in the DEAD-box protein family. Phylogenetic analysis placed SaVasa closest to the Vase subfamily within the DEAD-box family, rather than the PL10 and P68 subfamilies. These findings suggested that *Savasa* is a member of the Vasa homologues with a high degree of conservation during evolution. Additionally, reports have indicated the presence of variable splicing variants of *vasa* [13]; further research is needed to determine if splicing variants also exist in Savasa.

### 4.2. The Spatio-Temporal Expression Pattern of Savasa

Previous studies have examined the embryonic expression of *vasa* mRNA in some teleosts, including the zebrafish [8], grass carp (*Ctenopharyngodon idella*) [42], catfish (*Clarias gariepinus*) [45], Japanese flounder (*Paralichthys olivaceus*) [46], black carp [15], and Nile tilapia (*O. niloticus*) [47]. Generally, *vasa* mRNA is thought to be maternally supplied and is abundant in the early stages of embryos. For example, in black carp, the *Mpvasa* gene is maternally inherited and exhibits high expression levels from the unfertilized egg through the blastula stage, decreasing sharply thereafter. In grass carp, *vasa* transcript levels are high during cleavage and blastula stages, continuing to decrease until the gastrula stage. In the present study, *Savasa* mRNA was abundant in early embryos up to the blastula stage, then decreased sharply at the gastrula stage, followed by a gradual decline through the neurula and heart-beating stages, displaying a similar expression pattern to other *vasa* homolog in teleosts. This higher expression of *Savasa* during early embryogenesis may be attributed to the degradation of maternal *vasa* mRNA in later stages or a reduction in the proportion of PGCs due to an increase in the total number of embryonic cells.

The subcellular localization of *Savasa* was further detected by WISH in this study (Figure 5). As a key component of the germplasm, *vasa* plays a crucial role in designating PGCs and their migration route to the gonads in various fish species [15,26,48,49]. The WISH results in our study effectively highlighted the origin and migration of PGCs in *S. asotus*. Initially, *Savasa*-positive signals were observed at the cleavage furrow’s edge during the two-cell stage, appearing as two symmetrically distributed clusters, in accordance with the concept of ‘preformation’, where maternal reproductive material is localized in specific cells at the start of embryonic development. Similar patterns were noted in zebrafish, *Caenorhabditis elegans*, *Drosophila* and *Xenopus* [8,50,51]. Along with the development of embryos, the *Savasa* signals increased to four clusters at the four-cell stage, as the distribution of *vasa* in zebrafish [8]. The pattern persisted with four clusters in blastomers during the blastula stage. However, it grew more than four clusters during the following gastrula stage, which is in agreement with that in black carp but different from that in medaka. In medaka, *vasa* mRNA is widely distributed in early embryonic stages, then concentrated in PGCs until the gastrula stage [9]. This suggested that the distribution of PGCs varied among fish species despite the ‘preformation’ model. As for the migration of PGCs, our result showed that it migrates towards the dorsal side and forms two rows along the dorsal body axis, with an increase in *Savasa*-positive cells during the gastrula and subsequent stages. This dynamic expression of *Savasa* mRNA reflects the migration route of *vasa*-labeled PGCs in various fish [15,52], indicating *Savasa* as a potential marker gene for PGCs during early embryonic development.

The expression levels of *vasa* in different tissues of *S. asotus* were also detected using qRT-PCR. The findings noted a conserved expression pattern of *Savasa* in the gonads, predominantly in the ovary, suggesting a sexually dimorphic trend during development, consistent with the results observed in European sea bass (*Dicentrarchus labrax*) [53] and turbot (*Scophthalmus maximus*) [54]. However, there are some distinctions between them. In European sea bass, females consistently exhibited higher *vasa* expression levels compared to males in early development and sex differentiation stages. Conversely, in turbot, females displayed significant elevated *vasa* expression levels prior to sexual maturity, with a reversal in mature testes where *vasa* expression surpassed those in ovaries. These studies suggest different roles of the *vasa* gene during gonadal morphogenesis in different fish species.

Despite its gonadal expression pattern, *Savasa* was also examined in extra-gonadal tissues, as observed in species like *Xenopus* [3], the rainbow trout [44], the turbot [54] and the Senegalese sole (*Solea senegalensis*) [55]. Future investigation should be carried out to explore the possible mechanism of *Savasa* in extra-gonadal expression. Moreover, the subcellular localization of *Savasa* in the gonad was checked by FISH, revealing its restricted expression in germ cells of both female and male individuals, in accordance with observations in other teleosts [12,56,57]. Particularly, the gonadal *Savasa* mRNA was specifically found in spermatogonia and spermatocytes, similar to *vasa* expression in goldfish [58], mainly located in the most peripheral region and existing in fewer numbers compared to spermatids and sperms in the mature testis. Consequently, the lower expression of *Savasa* in testis, as detected by qRT-PCR, may be associated with the lower proportion of spermatogonia and spermatocytes in mature testis, as evidenced by FISH results. The *Savasa* expression in germ cells of the ovary and testis also indicates its potential role in gametogenesis and its utility as a gonadal germ cell marker in *S. asotus*.

### 4.3. Response of Savasa to Letrozole Treatment and the Possible Primary Mechanism

Previous studies have shown that applying exogenous substances like LT to fish during the critical stage of sex differentiation can lead to sex reversal [35,59,60]. The key period for sex differentiation in *S. asotus* is reported to be between 8 dph and 40 dph [36]. Nevertheless, the specific changes in *vasa* expression during this key period, especially at or after LT treatment, are not well understood. In this study, fish aged 8 dph to 40 dph were treated with different concentrations of LT following methods described by previous researchers to observe the response of *Savasa* [36]. The results noted that *Savasa* expression levels increased with the increasing LT concentrations, reaching the peak at 50 mg/kg at 40 dph during the critical period. And it was significantly raised at 40 dph compared to 8–24 dph with the same LT concentration. This pattern is similar to previous findings in the protandrous black porgy (*Acanthpagrus schlegeli*), in which *vasa* gene expression was stimulated by steroid and LT injections [61]. Since sexual differentiation is almost completed at 40 dph in *S. asotus*, the peak levels of *Savasa* mRNA at this stage suggest the gene is an important partner in sexual differentiation, and LT has a potential influence on this gene. Furthermore, studies have shown that *vasa* plays a role in the piRNA biosynthesis pathway, providing helicase activity for piRNA synthesis or silencing of reverse transcriptional transposons [62]. Disrupting sex hormone levels can activate the piRNA pathway in early loach fry development [63]. Exposure to falconazole can also upregulate *vasa* gene expression in the loach fry and *X. laevis*, and it is associated with an enhanced piRNA pathway [64]. In this study, LT, as a substance that inhibits estrogen biosynthesis, meant its administration led to an upregulation of *vasa* expression levels in *S. asotus*, which might also be related to the enhanced piRNA pathways. However, further research is necessary to verify this.

In this experiment, *Savasa* was detected in trace amounts in the brain of adult fish, consistent with previous findings [55]. This might suggest a potential link between *vasa* and RNA metabolism processes in the brain. Moreover, in female fish, after being treated with 50 mg/kg of LT, the expression of *Savasa* in both the brain and ovary decreased at 120 dpf, indicating inhibition of its expression during this stage. However, 20 mg/kg of LT appeared to stimulate *Savasa* expression in the brain, implying there is a threshold range of LT concentration on its influence on the gene in the female. In male fish, the expression levels of *Savasa* in the brain and testis were generally lower at 120 dph compared to the earlier time points, with the highest concentration group showing the most significant decrease. This decrease may be due to a shift in testicular cell composition and increased sperm production at 120 dph, as reported on the study that the expression level of the *vasa* gene tends to decrease gradually with gonadal maturation [54,65], suggesting that *Savasa* plays an important role in early spermatogenesis stages, which is in agreement with research on goldfish [58]. Differences on the response of *Savasa* to LT between the female and male imply the function and quantity of the gene vary in different sex of fish. And the changes in *Savasa* expression in brain and gonads after LT treatment might also relate to the interaction of the hypothalamic–pituitary–gonadal axis [66].

## 5. Conclusions

This study cloned *Savasa* from *S. asotus*, providing insights into its gene structure, protein alignment, phylogenetic tree, expression pattern, and subcellular localization in embryos and the gonads for the first time. The results suggest that *Savasa* is the ortholog of mammalian and teleost *Vasa*, maternally provided, and specifically expressed in germ cells, indicating the PGCs’ origin and migration in embryos and its distribution in the gonads. Furthermore, *LT treatment* during the key sex differentiation period could alter the expression levels of *Savasa* in this period and subsequent stages from 60 dph to 120 dph, and the responses of *Savasa* to LT treatment between the female and male have a certain degree of difference. This study establishes *vasa* as a potential marker for germ cells in *S. asotus*, indicates its possible roles in embryogenesis and gonadal development process, laying the foundation for future utilization and function research of this gene.

## Figures and Tables

**Table 1:** Primers used in this study.

Name	Sequence (5′-3′)	Usage
Sa-actin-F	AAGATCATTGCCCCACCTGA	qRT-PCR
Sa-actin-R	CCTGCTTGCTGATCCACATC	qRT-PCR
Sa-q-vasa-F	CCGAGAAGTGTTGAAAGGTT	qRT-PCR
Sa-q-vasa-R	CCAGCACCAAATAACGAACT	qRT-PCR
Sa-vasa-F	CTATCTTCAACTATGGAGGACTGG	ORF amplification
Sa-vasa-R	CAGTTTCCTCCAGCCATGAAG	ORF amplification
Sa-v-GSP	ACATCAATCAAGTCACTCGGTA	3′RACE PCR
Sa-v-NGSP	CCTACCGAAATACATTGACGAG	3′RACE PCR
Sa-vasa-situ F	AAAGAATTCGAACTCTTGGAACAACGGGAA	probe synthesis
Sa-vasa-situ R	AAATCTAGAGTAAATCCTGTGCTTGTACCTC	probe synthesis

## Data Availability

The original contributions presented in the study are included in the article/Supplementary Material, further inquiries can be directed to the corresponding author.

## Supplementary Materials

The following supporting information can be downloaded at: https://www.mdpi.com/article/10.3390/genes15060756/s1, Figure S1: mRNA detection using *Savasa* sense probe as a control during embryonic development.
