# Supplementary material for: Characteristics of the Vasa Gene in Silurus asotus and Its Expression Response to Letrozole Treatment
Source: Genes (Basel). 2024 Jun 8;15(6):756. doi: 10.3390/genes15060756 (PMC11202796; doi:10.3390/genes15060756)
Supplement: Supplementary file 1 [file genes-15-00756-s001.zip › genes-3020973-supplementary.pdf]

genes

MDPI

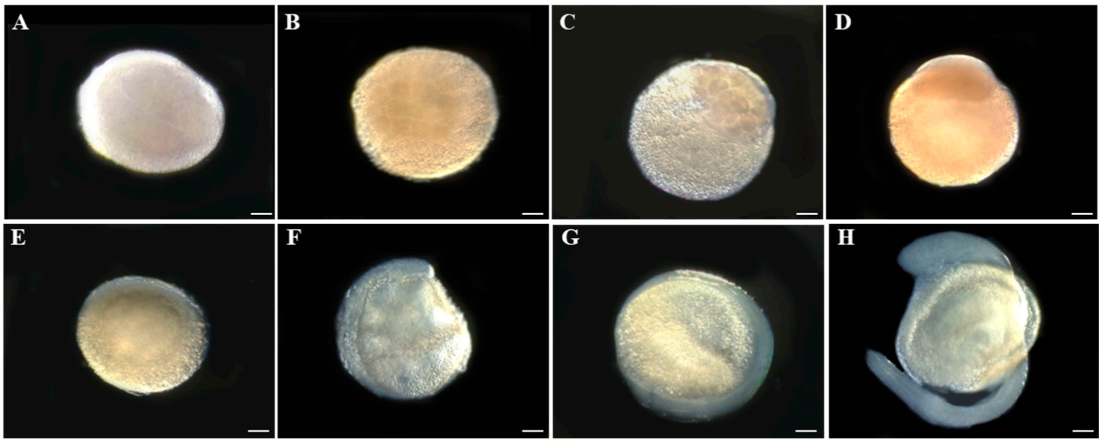

Figure S1. mRNA detection using *Savasa* sense probe as a control during embryonic development. The sense probe did not give any detectable signal during the developmental stages. (A) 2-cell; (B) 16-cell; (C) multi-cell; (D) blastula; (E) gastrula; (F) eye vesicle stage; (G) tail bud stage; (H) hatching stage. Scale bars=200 μm.
